# Effect of Colesevelam on Liver Fat Quantified by Magnetic Resonance in Nonalcoholic Steatohepatitis: A Randomized Controlled Trial

**DOI:** 10.1002/hep.25731

**Published:** 2012-07-02

**Authors:** Thuy-Anh Le, Joshua Chen, Christopher Changchien, Michael R Peterson, Yuko Kono, Heather Patton, Benjamin L Cohen, David Brenner, Claude Sirlin, Rohit Loomba, for the San Diego Integrated NAFLD Research Consortium (SINC)

**Affiliations:** 1 Division of Gastroenterology, Department of Medicine,Department of Family and Preventive Medicine, University of CaliforniaSan Diego, La Jolla, CA; 2 Department of Radiology,Department of Family and Preventive Medicine, University of CaliforniaSan Diego, La Jolla, CA; 3 Department of Pathology,Department of Family and Preventive Medicine, University of CaliforniaSan Diego, La Jolla, CA; 4 Division of Epidemiology, Department of Family and Preventive Medicine, University of CaliforniaSan Diego, La Jolla, CA

## Abstract

Bile acid sequestrants (BAS) lower plasma low density lipoprotein levels and improve glycemic control. Colestimide, a BAS, has been claimed by computed tomography to reduce liver fat. Therefore, we examined the efficacy of colesevelam, a potent BAS, to decrease liver fat in patients with biopsy-proven nonalcoholic steatohepatitis (NASH). Liver fat was measured by a novel magnetic resonance imaging (MRI) technique, the proton-density-fat-fraction (PDFF), as well as by conventional MR spectroscopy (MRS). Fifty patients with biopsy-proven NASH were randomly assigned to either colesevelam 3.75 g/day orally or placebo for 24 weeks. The primary outcome was change in liver fat as measured by MRI-PDFF in colocalized regions of interest within each of the nine liver segments. Compared with placebo, colesevelam increased liver fat by MRI-PDFF in all nine segments of the liver with a mean difference of 5.6% (*P* = 0.002). We cross-validated the MRI-PDFF-determined fat content with that assessed by colocalized MRS; the latter showed a mean difference of 4.9% (*P* = 0.014) in liver fat between the colesevelam and the placebo arms. MRI-PDFF correlated strongly with MRS-determined hepatic fat content (*r*^2^ = 0.96, *P* < 0.0001). Liver biopsy assessment of steatosis, cellular injury, and lobular inflammation did not detect any effect of treatment. *Conclusion:* Colesevelam increases liver fat in patients with NASH as assessed by MRI as well as MRS without significant changes seen on histology. Thus, MRI and MRS may be better than histology to detect longitudinal changes in hepatic fat in NASH. Underlying mechanisms and whether the small MR-detected increase in liver fat has clinical consequences is not known. (Hepatology 2012;56:922–932)

Nonalcoholic steatohepatitis (NASH) is a severe form of nonalcoholic fatty liver disease (NAFLD) that is characterized by features of steatosis, hepatocellular injury, and parenchymal inflammation with or without perisinusoidal fibrosis on liver histology in individuals who consume little or no alcohol.^1^ A subset of patients with NASH may progress to develop cirrhosis and hepatocellular carcinoma.^2^ Currently, there are no Food and Drug Administration (FDA)-approved treatments for NASH.

Colesevelam, a bile acid sequestrant (BAS), interrupts the enterohepatic circulation of bile acids and causes increased conversion of hepatic cholesterol to bile acids. Colesevelam increases plasma glucagon-like-peptide-1 (GLP-1) levels, which activates pancreatic β-cells to release insulin. One possible mechanism by which colesevelam causes increased GLP-1 is by moving the site of fat absorption to the ileum where fat absorption by L cells causes GLP-1 release.^3^ This might explain the mechanism of action of colesevelam that may be responsible for improving glucose homeostasis. Furthermore, randomized controlled clinical trials have shown the efficacy of colesevelam in lowering plasma low density lipoprotein cholesterol (LDL) levels and improving glycemic control.^4^-^6^ Both increased plasma LDL cholesterol and insulin resistance are associated with NASH. Therefore, it is plausible that improvement in LDL cholesterol and glycemic control by colesevelam might improve NASH. We hypothesized that colesevelam would reduce hepatic fat content in patients with NASH by increased utilization of hepatic cholesterol in bile acid synthesis and depletion of the bile acid pool by the BAS properties of colesevelam. Another BAS formulation, colestimide, was shown in a small open-label trial of Japanese patients with NAFLD to reduce liver fat as measured by computed tomography (CT).^7^

CT scanning is limited in its ability to accurately quantify liver fat. However, magnetic resonance imaging (MRI) provides a better assessment and accurate quantification of liver fat content. Recently, newer MRI has emerged as a useful modality that may be more accurate for the assessment of liver fat as compared to ultrasound, CT, and conventional MRI.^8^, ^9^ Using the conventional Dixon in- and out-of-phase (IOP) method, calculated MRI fat fraction was shown in previous studies to correlate well with hepatic steatosis.^8^, ^10^-^12^ The new development of the MRI-determined proton-density-fat-fraction (PDFF) technique further improves upon the Dixon IOP method by minimizing T_1_ bias and correcting T_2_* decay. This technique models the fat signal as a superposition of multiple frequency component interference to give a quantitative, standardized, and objective MRI measurement of hepatic fat content. Using MR spectroscopy (MRS) as a reference standard, PDFF has been shown to be an accurate, precise, and reproducible noninvasive method to assess hepatic steatosis.^13^-^15^ Furthermore, the novel PDFF MRI technique allows fat mapping for the entire liver, where longitudinal individual segment changes of liver fat can be accurately quantified and small differences can be detected, whereas MRS only measures liver fat content in a small region of interest. MRI-PDFF is an imaging-based technique as opposed to MRS, which is a biochemical-based technique that provides data on the liver fat content in a small region of interest in the liver. The aim of this study was to determine if colesevelam reduces liver fat as measured by MRI-PDFF in patients with biopsy-proven NASH in a randomized, placebo-controlled clinical trial.

## Patients and Methods

### Study Design

This was an investigator-initiated, randomized, double-blind, allocation-concealed, placebo-controlled clinical trial. This study was designed and conducted according to the CONSORT Guidelines to evaluate the efficacy of 3.75 g colesevelam taken orally daily versus identical-appearing placebo for 24 weeks in reducing liver fat in patients with NASH. The CONSORT checklist is available as Supporting Table 1. There were no changes to the methods or trial outcomes after trial commencement. No interim analysis was performed. The study patient population was derived from the San Diego Integrated NAFLD Research Consortium (SINC) cohort, which is a city-wide collaboration set up to study NAFLD led by the principal investigator (PI; R.L.), including University of California at San Diego (UCSD) Medical Center, Sharp Health System, Balboa Naval Medical Center, and Kaiser Permanente Southern California. Patients with biopsy-proven NASH seen at any of these sites were referred to the UCSD NAFLD research clinic. The study was conducted at the General Clinical Research Center (now called the Clinical and Translational Research Institute), UCSD. This study was registered at clinicaltrials.gov (registration number NCT01066364) and approved by the FDA under an IND to the PI (R.L.). The protocol was approved by the UCSD Institutional Review Board. Written informed consent was obtained from all patients.

### Patient Population

Patients were screened with history, physical examination, and all underwent an alcohol history assessment by completing the AUDIT and Skinner Lifetime Drinking questionnaire. Patients were asked to stop any medications being used for their liver disease including vitamins and herbal medications.

### Inclusion and Exclusion Criteria

Inclusion criteria included age 18 years or older, having serum alanine (ALT) or aspartate (AST) aminotransferase activity above the upper limits of normal: 19 U/L or more for women and 30 U/L or more for men, and presence of hepatic steatosis of >5% by MRI-PDFF. All patients had liver biopsies with evidence of NASH on biopsy performed within 6 months of enrollment without any significant change in body weight and interventions for treatment of NASH between the date of liver biopsy and enrollment. All patients with definite NASH as defined by the presence of all of the following features including steatosis, ballooning degeneration, and lobular inflammation with or without fibrosis as well as those with features suspicious for NASH defined as an NAFLD activity score (NAS) of 4 or higher were eligible. NAS ranges from 0-8 and is a sum of steatosis score (0-3), lobular inflammation score (0-3), and ballooning degeneration score (0-2). Exclusion criteria: Subjects were excluded if they had evidence of other forms of liver disease shown by the presence of serum hepatitis B surface antigen (HBsAg), hepatitis C viral RNA (HCV RNA), positive autoimmune serologies, evidence for hemochromatosis by 3+ or 4+ stainable iron on biopsy or homozygosity on genetic analysis, low ceruloplasmin levels with biopsy suggestive of Wilson's disease or low alpha-1-antitrypsin levels with biopsy suggestive of alpha-1-antitrypsin disease. Exclusion criteria also included alcohol intake of more than 30 g per day in the previous 10 years or greater than 10 g per day in the previous year, decompensated liver disease with Child-Pugh score greater than 7 points, active substance abuse, significant systemic illnesses, renal insufficiency (creatinine >1.5 mg/dL in men, and >1.4 mg/dL in women), positive human immunodeficiency virus (HIV) test, pregnancy, evidence of hepatocellular carcinoma (alpha-fetoprotein >200 or liver mass on imaging), ingestion of drugs known to cause hepatic steatosis, contraindications to liver biopsy (platelets <70,000/mm^3^ or prothrombin time >16 seconds), or inability to undergo MRI.

### Sample Size Estimation

*A priori,* we assumed that a 5% difference would be the minimal appreciable difference that may be clinically relevant. Based on the previous colestimide study, the colesevelam group was predicted to have at least a 6% reduction in liver fat compared to baseline, and 1% or less in the placebo group. We also predicted less than a 10% dropout based on previous studies conducted by the PI in NASH. To achieve a power of 90% with a β of 0.05, a sample size of 22 subjects in each arm was needed. Therefore, we planned to randomize a total of 50 patients to either colesevelam or placebo so that even with dropouts the study would be adequately powered to test the hypothesis.

### Baseline Assessments

Baseline evaluation prior to treatment initiation included routine history and physical exam, height, weight measurements performed by a trained investigator, and calculation of body mass index (BMI). Subjects underwent blood tests that included: ALT, AST, alkaline phosphatase, gamma-glutamyl transferase (GGT), total bilirubin, albumin, hemoglobin A1c, fasting glucose and insulin, prothrombin time/INR, lipid panel, and C-reactive protein (CRP).

### Liver Histology Protocol

Liver biopsy performed within 6 months of the start of the study were used as baseline liver biopsy; otherwise, a percutaneous liver biopsy was performed and was scored using the Nonalcoholic Steatohepatitis-Clinical Research Network (NASH-CRN) histologic scoring system. A single hepatopathologist (M.P.), who was blinded to clinical as well as radiologic data, the order of liver biopsy specimens, and previous results of the biopsy, performed all liver biopsy assessment for this study. All biopsies were re-read after completion of the protocol. Blinding was not broken until after completion of all histologic as well as radiologic/clinical data collection procedures. The average (± standard deviation, [SD]) length of liver biopsy specimen was 16.9 mm (±3.3) and the average (±SD) number of portal tracts seen on the biopsy specimen were 10.6 (±2.7). These are similar to previously performed high-quality clinical trials in NASH and reflect good clinical practice at a large NAFLD clinical research site.

NAS, which is the sum of the degree of steatosis (0-3), lobular inflammation (0-3), and hepatocellular ballooning (0-2), was obtained in all patients. The NAS ranges from 0-8. A two-point drop in NAS score was considered improvement in liver histology (with no change in hepatic fibrosis) and was considered an exploratory endpoint. Liver fibrosis (stage 0-4) was scored using the NASH-CRN histologic staging system (16).

### Randomization and Allocation Concealment

Subjects were randomized by the investigational drug services (IDS) at UCSD using computer-generated numbers to either treatment or placebo groups in blocks of 4 in a 1:1 ratio. Independent IDS pharmacists dispensed either active or placebo treatment pills, which were identical in appearance. Pills were prepackaged in identical bottles, labeled according to the computer-generated randomization numbers, and delivered to the research clinic office by a courier. The allocation sequence was concealed from the research coordinators and all investigators including hepatologists, radiologists, and pathologist who assessed and enrolled subjects in the trial. Patients were scheduled for individual appointments in the research clinic to avoid any overlapping appointments to reduce the likelihood of patients interacting and discussing treatment efficacy or side effects while in the waiting room. Treatment allocation was unblinded only after the completion of entire study procedures including all posttreatment liver biopsy and MRI studies on all patients.

### Study Visits

Upon meeting inclusion and exclusion criteria and completing baseline evaluation, patients were randomized at time designated as week 0 to receive either colesevelam 3.75 g/day (six 675-mg tablets either once or twice daily depending on patient preference with meals) or placebo for a total of 24 weeks. Patients returned to the research clinic at weeks 4, 12, and 24 after randomization. At these clinic visits, routine blood tests were obtained, body weight and vital signs were recorded, and the number of pills was counted to document compliance. A physical exam and careful history of liver-related symptoms as well as possible side effects of colesevelam were also obtained at each visit. At the completion of 24 weeks of therapy with either colesevelam or placebo, patients underwent MRI and MRS, biochemical testing, and a liver biopsy. After stopping treatment, the subjects had an 8-week follow-up visit to monitor for possible late side effects of treatment.

### MRI and MRS

#### Rationale for Selection of MRI as the Tool to Assess Liver Fat

MRI was selected to measure the primary outcome because it is noninvasive, uses no ionizing radiation, provides objective and quantitative estimates of fat content throughout the entire liver, is more objective than ultrasound (which is operator-dependent and interpreted qualitatively), is more accurate than CT (which has limited grading accuracy and also utilizes ionizing radiation), is more practical to perform and provides greater spatial coverage than MR spectroscopy (which is technically difficult to perform and analyze, and evaluates liver fat content in only a single 2 × 2 × 2 cm^3^ cube [voxel] within the liver).

#### MRI Protocol

Baseline and posttreatment MRI to assess hepatic steatosis were performed utilizing a state-of-the-art MRI-PDFF technique. The PDFF is a standardized objective measure of liver fat content; the measurement is independent of scanner manufacturer, scanner platform, field strength, and other confounders that frequently corrupt fat content estimations made by conventional MRI techniques used in prior clinical trials in NASH. This technique utilizes a gradient echo sequence with low flip angle (FA) to minimize T_1_ bias, and it acquires multiple echoes at echo times at which fat and water signals are nominally in-phase or out-of-phase relative to each other. Data obtained at each of the echo times are passed to a nonlinear least-squares fitting algorithm that estimates and corrects T_2_* effects, models the fat signal as a superposition of multiple frequency components, and estimates fat and water proton densities from which the fat content is calculated. Minimization of T_1_ bias, correction of T_2_* effects, and modeling the fat signal as a superposition of multiple frequency components have all been shown to improve fat quantification accuracy and robustness. Using custom analysis software developed by the UCSD Liver Imaging Group, the mathematical model is applied pixel-by-pixel on the source images to generate parametric PDFF maps that depict the quantity and distribution of fat segment by segment throughout the entire liver.

#### Quality Control

The MRI-PDFF technique and the analysis software have been tested and refined in adult research subjects.^10^, ^14^ Compared with MRS, the MRI-PDFF accurately and precisely measures liver fat fraction; in a prospective clinical study of 110 subjects at 1.5T, linear regression analysis showed a slope of 0.98 and an intercept of <1% between MRI- and MRS-measured liver fat fraction.^10^ MRI-PDFF is precise; in 38 subjects imaged twice on the same day, the Pearson *r*-correlation coefficient between repeated measurements was greater than 0.99 with a percent-error of less than 1%.^10^

#### Detailed Fat Mapping of the Whole Liver

MR examinations were performed by experienced research MR technologists with expertise in the utilized procedures and analyzed, under the supervision of the radiology investigator (C.S.), by a single trained image analyst blinded to clinical data, treatment group allocation, histological data, and the order (baseline or follow-up) of each scan. Imaging PDFF was recorded in regions of interest (ROIs) ≈300 to 400 mm^2^ in area placed on the PDFF parametric maps, avoiding blood vessels, bile ducts, and artifacts. To assess longitudinal changes in fat content, three colocalized ROIs were placed in each of the nine liver segments (27 separate ROIs) on the baseline and follow-up MR exams. For each segment, the three PDFF measurements were averaged.

#### Internal Validation Using MRS

In this study, MRS in a single location (voxel) in each liver was performed as a reference standard for colocalized MRI-PDFF measurements. In order to validate the accuracy of the imaging PDFF estimates, three additional ROIs were placed on the PDFF maps in the same locations as the spectroscopic voxel (one through the middle third of the voxel, one through the superior third of the voxel, and one through the inferior third of the voxel), and these PDFF measurements were averaged (Fig. 3). MR spectra were analyzed offline by an MR physicist with expertise in liver spectroscopy.

#### Statistical Analysis

The chi-square (χ^2^) test was used for comparisons between categorical variables and paired *t* test was used to compare mean differences between continuous variables in the colesevelam versus placebo groups. The primary analysis was performed as an intention-to-treat analysis. Primary and secondary comparisons within treatment groups were calculated using paired *t* tests, two-tailed independent sampled *t* tests, or nonparametric tests as appropriate. Pooled within group Pearson's correlations were used to look at associations across groups. A two-tailed *P* ≤ 0.05 was considered statistically significant. Statistical analyses were performed using SPSS v. 19.

## Results

### Description of Population

**Fig. 1:**
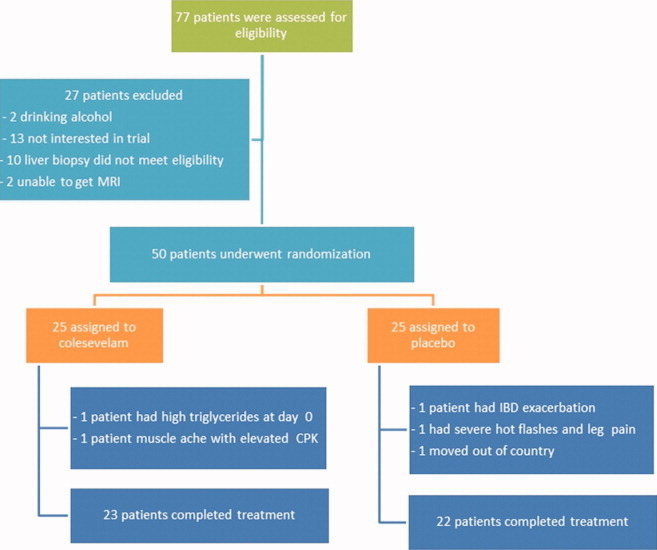
Chart of study enrollment and study flow. In all, 77 subjects were screened and 50 subjects were eligible for randomization. Twenty-five subjects were randomized to either colesevelam or placebo. There were two dropouts in the colesevelam and three dropouts in the placebo groups.

Between September 2009 and January 2011, 50 patients with biopsy-proven NASH were randomized to treatment with either colesevelam or placebo for 24 weeks. Of the 25 patients randomized to each group, two patients from the colesevelam and three patients from the placebo groups discontinued treatment (Fig. 1). The study population included 54% women. The ethnicity of the study population included 38% Caucasians, 28% Hispanic, 22% Asians, and 8% self-identified themselves as multiracial. The mean (±SD) age was 48 (±11.7) years. The mean (±SD) body-mass-index (BMI) was 31 (±4.8) kg/m^2^. Both groups had similar baseline characteristics, as shown in Table 1.

**Table 1:** Baseline Demographic, Biochemical, and Histologic Characteristics of Subjects

	Colesevelam (n=25)	Placebo (n=25)	*P-*Value
Demographics			
Male patients	10 (40%)	13 (52%)	0.571
Age (years)	45.4 (12.7)	50.3 (10.4)	0.140
Weight (kg)	89.6 (19.2)	87.0 (21.4)	0.643
Height (m)	1.7 (0.1)	1.6 (0.2)	0.255
BMI (kg/m^2)^	31.3 (4.7)	31.2 (5.1)	0.925
Ethnic origin:			
White	11 (44%)	8 (32%)	0.561
Black	0	0	—
Asian	3 (12%)	8 (32%)	0.171
Hispanic	8 (32%)	6 (24%)	0.754
Multiracial	2 (8%)	2 (8%)	1.00
Diabetes	8 (32%)	10 (40%)	0.769
Biochemical profile			
ALT	86.6 (68.1)	79.1 (47.8)	0.655
AST	56.2 (46.3)	49.9 (32.1)	0.582
AST:ALT	0.7 (0.2)	0.7 (0.3)	0.682
Glucose	105.0 (24.7)	112.5 (32.4)	0.358
Insulin	27.1 (40.2)	27.5 (27.1)	0.964
Hgb A1C	6.1 (0.8)	6.4 (0.9)	0.176
Triglycerides	198.0 (150.4)	166.5 (76.4)	0.355
Total cholesterol	200.2 (46.0)	202.4 (36.5)	0.852
LDL	124.0 (37.7)	115.8 (31.2)	0.405
FFA	0.5 (0.2)	0.5 (0.2)	0.404
Alk Phos	79.3 (26.1)	77.2 (18.6)	0.742
GGT	66.4 (38.3)	90.0 (83.5)	0.206
Total bilirubin	0.7 (0.5)	0.5 (0.2)	0.101
Direct bilirubin	0.1 (0.1)	0.1 (0.03)	0.102
Albumin	4.6 (0.3)	4.6 (0.3)	0.665
Protime	11.5 (0.7)	11.4 (0.7)	0.444
HOMA-IR	7.6 (12.3)	8.3 (9.8)	0.826
Histology			
Steatosis	1.9 (0.7)	2.2 (0.7)	0.247
Lobular inflammation	1.7 (0.7)	1.4 (0.7)	0.161
Ballooning	1.1 (0.7)	1.0 (0.6)	0.540
Fibrosis	1.2 (1.4)	1.1 (1.3)	0.840
NAS	4.7 (1.2)	4.6 (1.2)	0.729

BMI, Body Mass Index; AST, Aspartate Aminotransferase; ALT, Alanine Aminotransferase; Hgb A1C, hemoglobin A1C; LDL, Low-Density Lipoprotein; HDL, High-Density Lipoprotein; FFA, Free Fatty Acids; CRP, C-Reactive Protein; Alk Phos, Alkaline Phosphatase; GGT, Gamma-Glutamyl Transferase; HOMA, homeostatic model assessment; NAS, NAFLD Activity Score.

All labs were measured while fasting.

*T* test assuming equal variance between groups was performed on all continuous/ordinal variables and Fisher's exact test was performed on all categorical variables.

NASH-CRN histologic scoring system was used for histologic grading and staging of liver biopsy.

### Effect of Colesevelam on MRI-PDFF and MRS

**Fig. 2:**
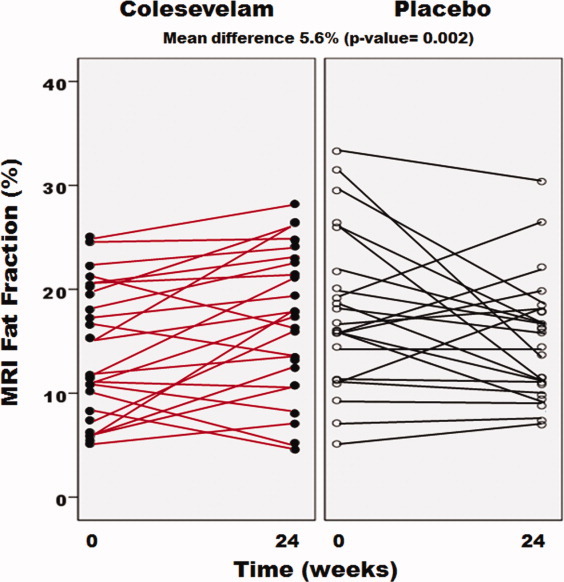
Effect of colesevelam on hepatic fat content assessed by MRI in patients with NASH. The longitudinal trend of liver fat fraction as measured by MRI-PDFF for each subject is shown in the placebo (right) and colesevelam (left) groups. Each small circle represents an individual patient at weeks 0 and 24. The average MRI-PDFF increased by 2.8% in the colesevelam group (*P* = 0.011) as shown by red lines and decreased by 2.7% in the placebo group (*P* = 0.065) as shown by black lines with a mean difference between the two groups of 5.6% (*P* = 0.002).

**Fig. 3:**
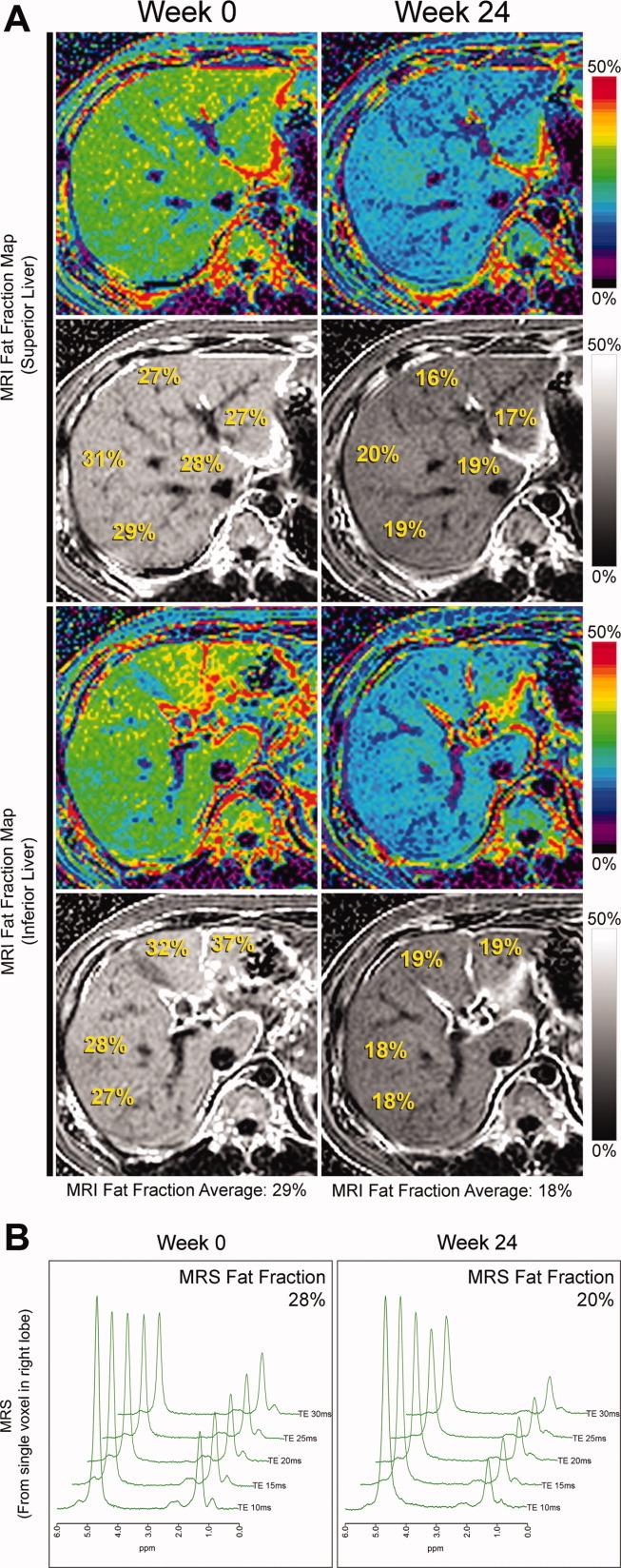
(A) Whole liver fat mapping with MRI-PDFF for a single patient. MRI-PDFF measurements of liver segments 1, 2, 4a, 7, and 8 in the superior plane (upper panel), and of liver segments 3, 4b, 5, and 6 in the inferior plane (lower panel) are shown at week 0 (left column) and 24 (right column) for a patient in the placebo group. The fat fraction in a single liver segment is calculated by averaging three MRI-PDFF ROIs. Using 27 ROIs, the calculated total liver fat fraction average at week 0 is 29% and this decreased to 18% at week 24. MRI-PDFF data from all nine liver segments gives a fat map for the entire liver where longitudinal within-segment changes of liver fat can be appreciated. (B) MRS measured fat fraction in the same patient. MRS measurements from a 2 × 2 × 2 cm^3^ cube (voxel) within the right liver lobe of the same patient in which MRI-PDFF were performed in (A) are shown at week 0 (left column) and week 24 (right column). The corresponding MRS fat fraction at week 0 is 28% and this decreased to 20% at week 24.

The colesevelam group had an unexpected increase in MRI fat fraction in all nine liver segments, as shown in Table 2. The total overall averaged MRI fat fraction increased from 14.2% to 17.0% (*P* = 0.01) in the colesevelam-treated patients. There was a trend toward a decrease in fat fraction in the placebo group from 17.9% to 15.2%. Compared with placebo, colesevelam increased liver fat using MRI-PDFF with a mean difference of 5.6%, which was statistically significant (*P* = 0.002). Individual patient data on changes in liver fat by MRI-PDFF stratified by the treatment group is shown in Fig. 2, confirming a small increase in the liver fat content in the colesevelam group and a small decrease in liver fat content in the placebo group. Detailed MRI-PDFF fat-mapping protocol of the nine liver segments and overall average at baseline and posttreatment at each level for a representative patient is shown in Fig. 3.

**Table 2:** Colesevelam Versus Placebo: Longitudinal Full Liver Fat Mapping Using Magnetic Resonance Imaging Proton Density Fat-Fraction (MRI PDFF) and Magnetic Resonance Spectroscopy (MRS) With Colocalized MRI Measurements

2A	Colesevelam (n=24)			Placebo (n=22)			Difference
			
Liver Segments	Baseline	Posttreatment	*P-*Value	Baseline	Posttreatment	*P-*Value	(*P-*Value)
1	12.5 (6.2)	15.1 (6.7)	**0.012**	16.5 (7.0)	14.2 (6.2)	0.114	**4.8 (0.005)**
2	13.0 (6.1)	15.6 (6.9)	**0.017**	17.2 (7.4)	14.3 (6.5)	0.060	**5.5 (0.003)**
3	13.9 (6.5)	16.8 (7.4)	**0.006**	18.4 (8.5)	15.2 (6.2)	0.055	**6.1 (0.002)**
4a	14.7 (6.4)	17.4 (6.9)	**0.008**	18.1 (7.7)	15.6 (6.1)	0.100	**5.2 (0.004)**
4b	14.7 (6.9)	17.5 (7.5)	**0.012**	18.1 (7.5)	15.6 (6.5)	0.082	**5.3 (0.003)**
5	14.5 (6.9)	17.5 (7.7)	**0.018**	17.7 (7.9)	14.9 (6.0)	0.062	**5.8 (0.003)**
6	14.0 (6.4)	17.1 (7.1)	**0.008**	17.9 (7.9)	15.3 (6.0)	0.082	**5.8 (0.002)**
7	15.0 (6.7)	18.0 (7.6)	**0.019**	18.6 (8.5)	15.4 (5.7)	**0.038**	**6.2 (0.002)**
8	15.4 (6.7)	18.1 (7.6)	**0.021**	18.8 (8.6)	15.9 (6.4)	0.058	**5.5 (0.003)**
MRI PDFF average	14.2 (6.3)	17.0 (7.0)	**0.011**	17.9 (7.7)	15.2 (6.0)	0.065	**5.6 (0.002)**

2B	Colesevelam (n=19)			Placebo (n=20)			Difference
			
	Baseline	Post-Treatment	*P-*Value	Baseline	Post-Treatment	*P-*Value	(*P-*Value)
MRS	16.8 (6.4)	18.7 (7.8)	0.106	18.4 (7.2)	16.1 (5.1)	0.127	**4.3 (0.028)**

2C	Colesevelam (n=20)			Placebo (n=21)			Difference
			
MRI-level	Baseline	Post-Treatment	*P-*Value	Baseline	Post-Treatment	*P-*Value	(*P-*Value)
MRI-s	14.8 (7.0)	17.5 (8.1)	0.065	17.4 (8.1)	15.2 (5.1)	0.167	**4.81 (0.016)**
MRI-m	15.2 (6.9)	17.3 (7.9)	0.089	17.7 (7.8)	14.5 (5.0)	0.089	**4.80 (0.017)**
MRI-i	14.9 (6.8)	17.1 (8.0)	**0.032**	17.5 (7.8)	14.5 (5.1)	0.080	**5.16 (0.013)**
MRI average	15.0 (6.9)	17.3 (8.0)	0.056	17.4 (7.9)	14.9 (5.0)	0.104	**4.9 (0.014)**
Pearson r (r^2^)	0.984 (0.968) *P=*0.000	0.996 (0.992) *P=*0.000		0.987 (0.974) *P=*0.000	0.961 (0.923) *P=*0.000		
Spearman ρ	0.978 *P=*0.000	0.995 *P=*0.000		0.985 *P=*0.000	0.952 *P=*0.000		

Data are expressed as means with standard error in parentheses or mean difference with *P-*value in parentheses.

Correlation coefficient expressed as Pearson coefficient r with r^2^ in parentheses and corresponding *P-*value, or as nonparametric Spearman's rho ρ with corresponding *P-*value.

MRI-PDFF, Magnetic Resonance Imaging Proton Density Fat Fraction; MRS, Magnetic Resonance Spectroscopy; MRI-s, Magnetic Resonance Imaging superior, MRI-m; Magnetic Resonance Imaging middle; MRI-I, Magnetic Resonance Imaging inferior.

Independent sample *t* test assuming equal variance was performed on all continuous variables for comparisons between groups. Paired-sample *t* test was performed for comparisons within group. Mean differences reflect comparison between baseline averages minus posttreatment averages.

2A: MRI PDFFs measured in all nine liver segments are used to calculate segmental and overall fat fraction averages at baseline and posttreatment between colesevelam and placebo group. There were three placebo and one Colesevelam dropout patients with no posttreatment MRI. Fat content in each liver segment are calculated by averaging three co-localized regions of interest (ROIs). The MRI total average is calculated using 27 ROIs, three from each liver segment.

2B: Longitudinal changes in MRS measurements from a 2x2x2 cm^3^ cube (voxel) within the liver at baseline and post-treatment in the colesevelam and placebo groups were used as a reference standard.

2C: Internal validation was performed by colocalizing MR imaging-based PDFF measurements to the reference MR-spectroscopy voxel. Three ROIs were placed on the PDFF maps in the same locations as the spectroscopic voxel (one through the superior third of the voxel[MRI-s], one through the middle third of the voxel[MRI-m], and one through the inferior third of the voxel[MRI-i]). PDFF measurements were averaged and correlation analysis to MR-spectroscopy measurements were performed.

To test the internal validity of MRI-PDFF findings, MRS (gold standard for hepatic fat quantification) was performed in colocalized ROIs with MRI-PDFF. MRS confirmed the results obtained by MRI-PDFF, with an increase in fat fraction in the colesevelam group and a decrease in the placebo group, with a mean difference of 4.9% (*P* = 0.014), as shown in Table 2. MRI-PDFF and MRS correlated robustly for measurements of fat fraction at baseline and posttreatment in both the colesevelam and placebo groups, with the correlation coefficient ranging from *r*^2^ = 0.96 to 0.98 (*P* < 0.0001).

### Effect of Colesevelam on Clinical and Biochemical Characteristics

As expected, patients treated with colesevelam had a significant reduction in LDL cholesterol by 18% (*P* = 0.001) and the difference was significant compared to placebo (*P* = 0.026) (Table 3). As colesevelam is known to reduce LDL cholesterol, this reduction in LDL provides an indirect measurement of compliance in both the groups. There were no significant changes in body weight or BMI between the two treatment groups. Compared to placebo, colesevelam group had a small increase in serum ALT (22.5 U/L, *P* = 0.084), alkaline phosphatase (8.1 U/L, *P* = 0.004), and GGT (35.4 U/L, *P* = 0.02). Otherwise, there were no significant differences between the groups in AST, insulin, glucose, hemoglobin A1C, HOMA-IR, triglycerides, total cholesterol, HDL, free fatty acids, bilirubin, albumin, and prothrombin time over a 24-week period in this patient population.

**Table 3:** Changes in Anthropometric and Biochemical Variable Between the Colesevelam- Versus Placebo-Treated Patients

	Colesevelam (n=25)			Placebo (n=23)			Difference
			
	Baseline	Posttreatment	*P-*Value	Baseline	Posttreatment	*P-*Value	(*P-*Value)
Weight (kg)	89.6 (19.2)	89.3 (18.9)	0.539	89.2 (20.8)	88.4 (19.9)	0.139	0.43 (0.605)
BMI (kg/m^2)^	31.3 (4.8)	31.2 (4.6)	0.545	31.7 (5.0)	31.3 (4.7)	0.128	0.25 (0.427)
ALT	86.6 (68.1)	109 (62.2)	0.084	78.9 (50.0)	65.2 (57.6)	0.052	**36.1 (0.016)**
AST	56.2 (46.3)	62.8 (33.7)	0.512	50.5 (33.5)	43.8 (36.0)	0.133	13.3 (0.238)
AST/ALT	0.7 (0.2)	0.6 (0.2)	0.075	0.68 (0.3)	0.72 (0.3)	0.167	**−0.10 (0.025)**
Glucose	105.0 (24.7)	102.8 (24.0)	0.444	113.6 (33.6)	112.9 (28.8)	0.903	−1.4 (0.826)
Insulin	27.1 (40.2)	32.7 (52.8)	0.243	28.5 (28.1)	32.5 (33.4)	0.588	−1.76 (0.831)
Hgb A1C	6.1 (0.8)	6.0 (0.8)	0.664	6.4 (1.0)	6.5 (1.0)	0.618	−0.12 (0.510)
Triglycerides	198.0 (150.3)	203.6 (73.8)	0.942	168.3 (76.7)	172.2 (88.5)	0.549	−4.75 (0.860)
Total Cholesterol	200.2 (46.0)	187.3 (35.1)	0.076	201.5 (37.7)	200.6 (43.2)	0.512	−12.7 (0.098)
LDL	119.1 (44.5)	106 (33.4)	**0.002**	116.7 (32.4)	111.8 (38.5)	0.241	**−15.33 (0.021)**
FFA	0.5 (0.2)	0.5 (0.2)	0.387	0.5 (0.2)	0.5 (0.2)	0.293	0.10 (0.190)
Alk Phos	79.3 (26.1)	87.4 (30.5)	**0.004**	77.5 (18.7)	76.7 (19.2)	0.624	**8.99 (0.006)**
GGT	66.4 (38.2)	99.5 (84.5)	**0.009**	84.7 (83.5)	87.4 (106.3)	0.665	**30.8 (0.028)**
Total Bilirubin	0.7 (0.5)	0.7 (0.5)	1.00	0.5 (0.2)	0.5 (0.2)	1.00	0.00 (1.00)
Direct Bilirubin	0.1 (0.1)	0.2 (0.1)	**0.047**	0.1 (0.0)	0.1 (0.0)	0.575	0.032 (0.109)
Albumin	4.6 (0.3)	4.6 (0.3)	0.643	4.6 (0.3)	4.6 (0.3)	0.648	0.00 (0.978)
Protime	11.5 (0.7)	11.5 (1.4)	0.913	11.4 (0.7)	11.3 (0.9)	0.390	0.085 (0.773)
HOMA-IR	7.6 (12.3)	8.4 (12.9)	0.609	8.7 (10.1)	10.0 (12.8)	0.542	−0.73 (0.776)

Data are expressed as means with standard error in parentheses or mean difference with *P-*value in parentheses.

BMI, Body Mass Index; AST, Aspartate Aminotransferase; ALT, Alanine Aminotransferase; Hgb A1c, hemoglobin A1c; LDL, Low-Density Lipoprotein; HDL, High-Density Lipoprotein; FFA, Free Fatty Acids; Alk Phos, Alkaline Phosphatase; GGT, Gamma-Glutamyl Transferase; HOMA, homeostatic model assessment**;** CRP, C-Reactive Protein.

Independent sample *t*test assuming equal variance was performed on all continuous variables for comparisons between groups. Paired-sample *t* test was performed for comparisons within group. Mean differences reflect comparison between baseline averages minus posttreatment averages.

### Liver Histology

Liver biopsy assessment did not detect any differences in steatosis grade between the two treatment arms (Table 4). Of the 31 patients with paired liver biopsy, 7/17 patients in the colesevelam group versus 2/14 patients in the placebo group showed a 2-point improvement in nonalcoholic fatty liver disease activity score (NAS, range, 0-8), which was not statistically significant (*P* = 0.1). The mean change in NAS did not differ between in the two treatment arms. Within-group comparisons showed that the small subgroup of colesevelam patients with paired liver biopsy had a mild improvement in the steatosis but this was not significant when compared to placebo. There were no significant differences in liver histology between the two treatment arms. The results remained consistent when MRI-PDFF and MRS were examined within this subgroup of 31 patients showing a small amount of increase in liver fat in the colesevelam group and decrease in the placebo group (data not shown).

**Table 4:** Changes in Liver Histology in Colesevelam- Versus Placebo-Treated Patients

	Colesevelam (n=17)			Placebo (n=14)			Difference
			
	Baseline	Posttreatment	*P-*Value	Baseline	Posttreatment	*P-*Value	(*P-*Value)
Steatosis							
Mean ± SE	2.00 (0.7)	1.53 (0.8)	0.021	1.86 (0.8)	1.50 (0.8)	0.096	0.598
Median ± IQR	2.0 (1.5-2.5)	1.0 (1.0-2.0)		2.0 (1.0-2.25)	1.5 (1.0-2.0)		
N grade 0/1/2/3	0/4/9/4	0/11/3/3		0/5/6/3	1/6/6/1		
Lobular inflammation							
Mean ± SE	1.82 (0.7)	1.59 (0.7)	0.248	1.43 (0.7)	1.71 (0.7)	0.102	0.069
Median ± IQR	2.0 (1.0-2.0)	1.0 (1.0-2.0)		1.0 (1.0-2.0)	2.0 (1.0-2.0)		
N grade 0/1/2/3	0/6/8/3	0/9/6/2		0/9/4/1	0/6/6/2		
Ballooning							
Mean ± SE	1.12 (0.8)	1.06 (0.7)	0.782	1.07 (0.7)	1.14 (0.7)	0.655	0.488
Median ± IQR	1.0 (0.5-2.0)	1.0 (0.5-2.0)		1.0 (0.75-2.0)	1.0 (1.0-2.0)		
N grade 0/1/2	4/7/6	4/8/5		3/7/4	2/8/4		
Fibrosis							
Mean ± SE	1.12 (1.5)	1.06 (1.3)	0.660	1.36 (1.6)	1.50 (1.4)	0.480	0.229
Median ± IQR	0 (0.0-3.0)	1.0 (0.0-2.0)		1.0 (0.0-3.0)	1.0 (0.0-2.25)		
Stage 0/1/2/3/4	9/3/0/4/1	8/4/2/2/1		6/3/1/2/2	4/4/3/1/2		
NAS							
Mean ± SE	4.89 (1.3)	4.18 (1.8)	0.151	4.36 (1.3)	4.36 (1.8)	0.927	0.136
Median ± IQR	5.0 (4.0-6.0)	3.0 (3.0-6.0)		4.0 (3.75-5.0)	4.0 (3.0-5.25)		

Data are expressed as means with standard error in parentheses or as median with interquartile range in parentheses.

SE, standard error; IQR, interquartile range.

Nonparametric tests (Wilcoxon-rank sum test) for related samples and independent samples were performed on all variables for histologic comparisons within groups and between groups respectively. Mean differences reflect comparison between baseline averages minus posttreatment values.

### Adverse Events and Compliance

Of the 25 patients in each treatment arm, two patients in the colesevelam group and three in the placebo dropped out of the study. In the colesevelam group, one patient discontinued treatment after 1 day of starting when baseline lipids revealed high triglycerides of 817 mg/dL and a second patient had medication discontinued when she developed diffuse muscle ache, which was not thought to be related to the study medication. In the placebo group, treatment was discontinued because one patient had exacerbation of his underlying inflammatory bowel disease shortly before week 12 of treatment; one had complaints of intolerable hot flashes, and one was lost to follow-up after moving out of the country because of employment. Overall, there were no differences in the side effects between the groups, as shown in the Supporting Table 2. The average compliance rate based upon pill count at each visit was 95%.

## Discussion

### Main Findings

In this randomized, placebo-controlled, clinical trial, treatment with colesevelam compared to placebo was associated with an unexpected increase in liver fat in patients with NASH as assessed by a novel and accurate MRI technique. The internal validity of the MRI-derived changes in liver fat were confirmed by colocalized MR spectroscopy-based measurements. The increase in liver fat could only be detected by MRI and spectroscopy but not by liver biopsy. This discrepancy is presumably because histology was not available on all the 50 patients but only 31 patients who underwent paired liver biopsy, with 17 patients in the colesevelam arm and 14 patients in the placebo arm. In addition, it likely is influenced by type 1 error due to multiple comparisons in the histology tables. Furthermore, the validity of the liver biopsy to detect changes in steatosis is limited due to sampling variability and subjective assessment on an ordinal rather than a linear (or continuous) scale. Liver biopsy lacks the precision to ascertain small (3%) but real differences in liver fat content that can be appreciated by MRI and spectroscopy. The patient population had very minimal fibrosis and overall low NAS at baseline, which may have potentially influenced the study results. Utilizing a randomized controlled study design, we demonstrate that MRI-based PDFF may be a better tool than liver histologic assessment to assess longitudinal changes in hepatic steatosis in the setting of a clinical trial. This MRI-PDFF technique can be performed on routine clinical MRI scanners.

### In Context With Published Literature

To our knowledge, this is the first randomized, double-blinded, placebo-controlled trial to evaluate the use of colesevelam in the treatment of NASH, and the second to study BAS in this patient population. The earlier open-label study by Taniai et al.^7^ showed that colestimide decreased BMI, hemoglobin A1c, AST, and hepatic steatosis as measured by CT-derived liver spleen ratio. Our study utilized a randomized, placebo-controlled design and had a larger sample size. Furthermore, we utilized MRI rather than CT because MRI is more accurate than CT for assessing liver fat. This study revealed that colesevelam, a different BAS, treated patients had an increase in MRI fat fraction with no significant change in body weight. Furthermore, our results are consistent with a previous study conducted by Davidson et al.^17^ that showed a small increase in serum ALT and alkaline phosphatase in the colesevelam-treated patients.

Although the results are contrary to our hypothesis and are opposite to the study done of Taniai et al., recent studies offer new insights to explain the effect of BAS on liver fat content and support the findings presented in this article. Brufau et al.^18^ showed that although colesevelam led to significant increases in fecal bile salt output, the total pool of bile salts remained unchanged and accounted for massively increased synthesis of bile salt cholate in the liver. In mice treated with colesevelam, there was an increase in bile salt loss paralleled by a robust compensatory increase in bile acid synthesis as well as fatty acid synthesis along with increased expression of lipogenic genes.^19^ Furthermore, colesevelam-treated mice had an increase in hepatic fat content, as seen in this human study. We conjecture that a similar phenomenon may occur in NASH patients treated with colesevelam in that bile acid sequestration may lead to a compensatory increase in bile acid synthesis and fatty acid synthesis in the liver, which is reflected by an increase in MRI fat fraction.

### Strengths and Limitations

We acknowledge the following limitations of our study. First, the results are contrary to the hypothesis and prior study by Taniai et al., which may suggest that there was insufficient compliance or power. However, the compliance was documented by pill count and was 95% in both groups. We also confirmed the LDL cholesterol-lowering effect of colesevelam compared to placebo as shown in previously published studies.^4^, ^18^, ^20^ Both of these provide independent confirmation of compliance and adherence to the assigned study group in the trial. Second, we were not able to obtain MRI studies on all of our patients. One patient in the colesevelam group and three patients in the placebo group did not receive end-of-treatment MRI for reasons explained in the Results section. However, given the overall changes in all nine liver segments, it is doubtful whether four additional studies would have changed the direction of results. Furthermore, the sample size estimates accounted for a 10% dropout rate. Third, the study was not powered to assess improvement or changes in liver histology. Histologic improvement in lobular inflammation, ballooning degeneration, and fibrosis are clinically significant endpoints that may be more important than steatosis alone, and these histologic parameters in addition to steatosis should be included in a NASH treatment trial. Liver histologic changes were not the primary outcome of the study because there were no prior published data to estimate effect size to adequately power the study and calculate sample size to examine improvement in liver histology. To our knowledge, this is the first pilot study of colesevelam in which liver biopsy has been performed.

The strengths of the trial include the randomized, placebo-controlled, double-blind design of the study. Allocation concealment and randomization technique and procedures have been described. Blinding was strictly enforced. Our study population was multiethnic, including a good proportion of non-Caucasians and had adequate sex-distribution, including 54% women.

### Innovation and Novelty: MRI Protocol for Clinical Trials

In this study we utilized a novel MRI-derived PDFF technique to perform fat mapping of the entire liver with detailed assessment of liver fat content in all nine segments of the liver before and after treatment. Further comparisons showed high correlation between MRI-derived PDFF and MRS-derived fat content. Segmental MRI-PDFF is a novel method that allows accurate quantification and localization of liver fat that is not feasible with current liver biopsy assessment. These data could also be utilized for determining sample size and estimating effect size in improving liver fat in weight loss studies in NASH/NAFLD.

In conclusion, this randomized, placebo-controlled, clinical trial showed that colesevelam may cause a small but measurable increase in liver fat in patients with NASH. This change in liver fat could only be detected by MRI. This observation indicates that MRI-PDFF may be a better tool than liver histologic assessment to quantify changes in hepatic steatosis in the setting of a clinical trial. The MRI-based technique used to measure PDFF is widely available and can be applied on any MR platform. Future NASH studies may utilize these novel MRI-based whole liver fat mapping techniques to better quantify longitudinal changes in liver fat.

## Abbreviations

BAS: bile acid sequestrants
MRI: magnetic resonance imaging
MRS: magnetic resonance spectroscopy
NASH: nonalcoholic steatohepatitis
PDFF: proton-density-fat-fraction
