# Supplementary material for: Effect of Colesevelam on Liver Fat Quantified by Magnetic Resonance in Nonalcoholic Steatohepatitis: A Randomized Controlled Trial
Source: Hepatology. 2012 Jul 2;56(3):922–32. doi: 10.1002/hep.25731 (PMC3400720; doi:10.1002/hep.25731)
Supplement: Supplementary file 2 [file hep0056-0922-SD2.doc]

**Supporting Table 2:** Adverse events in colesevelam and placebo groups.

| **Adverse events** | **Coleselevam** | **Placebo** | **P-value** |
| --- | --- | --- | --- |
| **General** |  |  |  |
| Fatigue | 3 | 1 | 0.609 |
| Allergies | 1 | 1 | 1.000 |
| Anxiety | 0 | 1 | 1.000 |
| Insomnia | 1 | 0 | 1.000 |
| **Gastrointestinal** |  |  |  |
| Bloating | 3 | 7 | 0.289 |
| Flatus | 4 | 2 | 0.667 |
| Diarrhea | 1 | 2 | 1.000 |
| Constipation | 2 | 4 | 0.667 |
| Heartburn | 1 | 1 | 1.000 |
| Abdominal discomfort | 4 | 6 | 0.725 |
| Ulcerative colitis flair | 0 | 2 | 0.490 |
| Nausea/vomiting | 1 | 1 | 1.000 |
| Dark stool | 1 | 0 | 1.000 |
| Blood in stool | 2 | 0 | 0.490 |
| **Dermatologic** |  |  |  |
| Increased facial hair | 0 | 1 | 1.000 |
| Rash | 0 | 2 | 0.490 |
| **Genito-urinary** |  |  |  |
| Urinary tract infection | 2 | 1 | 1.000 |
| **Otolaryngology** |  |  |  |
| Dry tongue | 0 | 1 | 1.000 |
| Nasal pain | 0 | 1 | 1.000 |
| **Cardiovascular** |  |  |  |
| Dizziness | 1 | 0 | 1.000 |
| Hypertension (*one was hypertensive crisis) | 2 | 0 | 0.490 |
| Vertigo | 1 | 0 | 1.000 |
| Atrial fibrillation | 0 | 1 | 1.000 |
| Shortness of breath | 0 | 1 | 1.000 |
| Leg edema | 0 | 1 | 1.000 |
| Chest pain | 1 | 1 | 1.000 |
| **Pulmonary** |  |  |  |
| Possible pneumonia | 0 | 1 | 1.000 |
| Asthma exacerbation | 0 | 1 | 1.000 |
| Upper respiratory infection | 3 | 2 | 1.000 |
| Cough | 0 | 2 | 0.490 |
| Sleep apnea worsening | 2 | 0 | 0.490 |
| **Musculoskeletal** |  |  |  |
| Fracture toe | 1 | 0 | 1.000 |
| Pain in hand or feet | 1 | 0 | 1.000 |
| Muscle aches/soreness | 2 | 3 | 1.000 |
| Restless leg syndrome | 1 | 0 | 1.000 |
| Back pain | 0 | 1 | 1.000 |
| Tendonitis | 0 | 1 | 1.000 |
| Joint pain | 1 | 1 | 1.000 |
| Sprain joint | 1 | 2 | 1.000 |
| **Ophthalmological** |  |  |  |
| Blurry vision | 0 | 1 | 1.000 |
| Retinal detachment | 1 | 0 | 1.000 |
| Stye-right eye | 0 | 1 | 1.000 |
| Conjunctivitis | 1 | 0 | 1.000 |
| **Infectious disease** |  |  |  |
| Tooth infection | 1 | 1 | 1.000 |
| Tooth ache | 1 | 0 | 1.000 |
| Flu | 1 | 0 | 1.000 |
| **Neurological** |  |  |  |
| Headache | 2 | 2 | 1.000 |
| Nerve-damage neck | 0 | 1 | 1.000 |
| Weakness of thigh | 0 | 1 | 1.000 |
| **Endocrine** |  |  |  |
| Worsening blood sugars | 1 | 1 | 1.000 |
| Feeling cold | 1 | 0 | 1.000 |
| New hot flash | 0 | 1 | 1.000 |

Footnote: Data is expressed as means with standard error in parentheses or numbers with percentage in parentheses.

Fisher’s exact test was performed on all categorical variables to calculate p-value
